# Correlates of Social Exclusion and Negative Labeling and Devaluation of People Living with HIV/AIDS in Rural Settings: Evidence from a General Household Survey in Zambézia Province, Mozambique

**DOI:** 10.1371/journal.pone.0075744

**Published:** 2013-10-11

**Authors:** Abraham Mukolo, Meridith Blevins, Bart Victor, Lara M. E. Vaz, Mohsin Sidat, Alfredo Vergara

**Affiliations:** 1 Vanderbilt Institute for Global Health, Vanderbilt University School of Medicine, Nashville, Tennessee, United States of America; 2 Department of Preventive Medicine, Vanderbilt University School of Medicine, Nashville, Tennessee, United States of America; 3 Department of Biostatistics, Vanderbilt University School of Medicine, Nashville, Tennessee, United States of America; 4 Friends in Global Health, Maputo, Mozambique; 5 School of Medicine, Universidade Eduardo Mondlane, Maputo, Mozambique; 6 Owen Graduate School of Management, Vanderbilt University, Nashville, Tennessee, United States of America; University of Buea, Cameroon

## Abstract

**Background:**

Increased HIV/AIDS knowledge and access to antiretroviral treatment (ART) have been hypothesized to decrease HIV stigma. However, stigma persists as a barrier to HIV services uptake. We studied the relationship between stigma, knowledge and attitudes towards HIV and its treatment, and confidence in the legal system (legal rights certitude).

**Methods:**

We analyzed data from a household survey of 3749 randomly sampled female heads of households in 259 enumeration areas across 14 districts of Zambézia Province, Mozambique. The questionnaire included questions about beliefs, attitudes and behavior towards PLWHA, HIV transmission knowledge, treatment-related beliefs, and legal rights certitude. Factor analysis distinguished two stigma constructs: Negative labeling and devaluation (NLD) and social exclusion (SoE). Multivariable linear regression was used to determine the association between stigma, knowledge of HIV/AIDS, treatment-related beliefs, and legal rights certitude, while controlling for variance in socio-demographics.

**Results:**

A 4-point increase in knowledge about HIV transmission was associated with more than a 3 unit decrease in NLD and SoE stigma scores (p<0.001). Given HIV transmission knowledge, a 25-point increase in legal rights certitude was associated with a 4.62 unit drop in NLD stigma (p<0.001); we did not detect an association between legal rights certitude and SoE stigma. Knowing at least one HIV positive person was associated with lower SoE (−3.17, 95% CI: −5.78, −0.56); no association with NLD (p = 0.1) was detected. ART efficacy belief was associated with higher NLD and lower SoE (2.90 increase and 6.94 decrease, respectively; p≤0.001).

**Conclusion:**

Increasing knowledge about HIV transmission and access to ART are likely to reduce stigma, but neither of the two is a panacea. Raising community awareness of the legal rights of PLWHA might improve the efficacy of stigma reduction efforts. Strategies that focus on specific domains of stigma might be more effective than generic stigma reduction strategies.

## Introduction

Stigma causes unnecessary suffering among people living with the human immunodeficiency virus/acquired immune deficiency syndrome (HIV/AIDS) [1]–[7]. This often undermines their capacity to access and utilize available healthcare and realize favorable health outcomes [8]–[11]. Hence, stigma reduction is also central to the long-term success of efforts to prevent and treat HIV/AIDS [12]. Understanding factors that account for the persistence of stigma in the age of antiretroviral treatment (ART) is important to inform stigma reduction efforts and has been advocated [13].

Stigma has been defined as a demeaning social evaluation or label that is attached to or tagged onto the entity or state of health that exhibits socially undesirable characteristics [2], [3], [17]. Stigmatization refers to the social process by which demeaning evaluations or labels and the consequent negative emotional and behavioral responses are generated and sustained [2]. The labeling theory of stigma posits that stigmatization is a sequential process that begins with negative labeling (based on perceived deviance from a given norm) and stereotyping of the deviant entity by others, which leads to separation and status loss (or devaluation) of the labeled entity, and subsequently social exclusion [14], [15]. This occurs in social contexts where the stigmatized have limited social power and legal protections against social harm [15], [16]. Whereas the state of health (e.g., HIV infection or HIV/AIDS disease) or entity (e.g., the person living with HIV) is not the stigma, certain attributes of the entity/health state trigger this negative social process [1]–[3]. Hence, HIV infection might be highly stigma-inducing in some socio-cultural contexts but not so stigma-inducing in other contexts.

One of the ways that literature classifies stigma is by the social context in which it is expressed/experienced [17]. This often equates to specifying who the stigmatizer is. Hence, community stigma, the focus of this study, refers to stigma held in the public mind, i.e., the general public or community stigmatizes HIV infection and/or people living with HIV/AIDS (PLWHA). Individuals in the community may endorse or refuse to endorse the negative attitudes and behaviors generally expressed in their community towards HIV infection and PLWHA. The impact of stigma on HIV services uptake might be assessed by relating individuals' services uptake to variance in the level of community stigma they endorse.

Stigma is sustained by a complex set of factors that are not easy to address [12], [17]. However, increased HIV/AIDS knowledge and availability of (or access to) ART have been hypothesized to diminish community stigmatization of people living with HIV/AIDS (PLWHA) [18], [19]. This should be most evident in resource limited settings, where the biomedical health system is less well established but is being rapidly scaled-up. In such settings, the public learns about HIV transmission routes, risk of HIV infection associated with everyday conduct, and the efficacy of HIV prevention and treatment measures from public health education programs. The scale-up of ART, to the extent that it changes the image of HIV disease from a life threatening condition (extremely stigmatizing due to existential threat [20]) to a chronic illness (potentially less stigmatizing and hope-inducing due to the probability of clinical remission and enhanced quality of life) [18] should impede the negative effects of stigma. Hence, knowledge of ART-experienced patients who live successfully with HIV/AIDS and/or belief in the efficacy of ART should correlate negatively with community stigma. However, stigma persists as a barrier to HIV service uptake world-wide [12], [19], [21]–[30]. The limitations of ART scale-up as a stigma reduction strategy have been noted in diverse settings [13], [25]. The advantages of investigating stigma under varying contexts of HIV knowledge and public health response to the epidemic have also been suggested [31]. The interaction effects of knowledge about HIV transmission and perceived efficacy of ART (both potential outcomes of ART scale-up) on willingness to endorse community stigma have also not been clearly described.

Human rights education has been proposed as another way to reduce stigma, especially in developing country settings where there are low levels of awareness of universally accepted human rights and inadequate legal statute defining and protecting the rights of PLWHA [3], [21], [32]. Even when knowledge of HIV-specific legal protections might be limited (a likely state of affairs in rural settings), generalized legal rights certitude could influence willingness to endorse community stigma, independent of knowledge about HIV transmission and treatment. In this case legal rights certitude refers to confidence in the legal system, in terms of one's ability to access the legal system and be guaranteed due process when need arises, regardless of the legal question at hand. However, the impact of legal rights certitude (or knowledge) on HIV stigma has not been adequately described.

Zambézia Province in Mozambique is one context where the HIV epidemic has had a profound impact at multiple levels including attracting significant investments in health promotion and HIV care and treatment programs [33]–[36]. Zambézia Province is the second most populated province of Mozambique. In 2007 about 3 million people lived in Zambézia Province, of whom over 86% resided in rural areas [33]. According to the 2009 antenatal sentinel surveillance of pregnant women aged 15–49, Zambézia has an HIV prevalence of 12.6% compared to the national prevalence estimate of 11.5% [36]. Despite high prevalence, Zambézia Province, and indeed most of Mozambique, has experienced some decline in prevalence of HIV since it peaked in 2005 at about 19% [36]. These trends are largely attributed to the scale-up of ART services [33], [37], [38]. Legal protections against discrimination on the grounds of HIV status have been instituted in Mozambique [39], consistent with global trends to reduce stigma through strategies that protect the dignity and human rights of PLWHA [12]. However, stigma is one of the negative factors consistently captured in studies about patient loss to follow-up and inadequate adherence to ART in Zambézia Province [40], [41] and other provinces in Mozambique [5], [34], [42]. Zambézia Province and Mozambique are not the only ART expansion zones where stigma persists as a barrier to healthcare and ART uptake [3], [6], [7], [10], [24], [43]–[52]. However, few studies have described the nature of community stigma in Zambézia Province and its correlates.

In this paper, we describe the way a population sample of female heads of households in Zambézia Province endorsed community stigma towards PLWHA and factors associated with these endorsement patterns. We specifically examine the association between stigma endorsement, legal rights certitude, knowledge about HIV transmission, familiarity with HIV infection, and beliefs about the efficacy of treatments for HIV/AIDS (See Table 1 for specific hypotheses and their respective rationale). While national demographic household surveys (DHS) have assessed public attitudes and knowledge about HIV transmission in Zambézia Province [37], literature on correlates of community stigma is scarce. The few reports about stigma have so far emerged from either very small surveys of convenient samples or only captured the views of HIV patients and healthcare providers in restricted geographic areas of the Province [40], [41]. We also highlight implications that the observed relationships might have for the design of anti-stigma interventions.

**Table 1:** Main hypotheses and related rationale*.

Hypothesis	Theory or potential causal mechanism
High knowledge of HIV transmission is **associated with low endorsement of stigma**	Prejudice theory. HIV stigma is related to ignorance or miss-information about HIV infection and its mode of transmission.
Awareness of HIV infection in self, friends **or relatives is associated with low stigma endorsement**	Othering and social proximity theories. Self identification or intimate relatedness with a socially devalued entity or state of health moderates negative affect towards the entity or state of health. Any person living with HIV/AIDS (and/or HIV infection itself) acquires an insider identity with the HIV-infected observer and/or the observer who is intimately related to another HIV-infected person.
Perceived risk of HIV infection will be **associated with low endorsement of stigma**	Othering and social proximity theories. Self identification or intimate relatedness with a stigmatized entity or state of health moderates negative affect towards the entity or state of health. The stronger the perceived risk of infection with HIV, the stronger the self-identification with people living with HIV/AIDS (PLWHA).
Believe in the efficacy of HIV/AIDS **treatment or the treatability of HIV/AIDS will be associated with low stigma endorsement**	Existential anxiety theory holds that a belief in the controllability of a life threatening illness or state of health moderates anxiety about and/or fear of the illness or health state.
High legal rights certitude is associated with **low** endorsement of stigma	Knowledge of legal rights and confidence in the legal system's capacity to protect all persons from harm is likely to moderate harmful attitudes and behavior towards PLWHA. The legal system can influence community stigma by dictating acceptable and unacceptable conduct and expressions, means of redress available to victims of stigma, and punishments for offenders.
HIV transmission knowledge will interact **with distance from and contact with health services to determine the level of stigma endorsed by participants**	Information diffusion theory. Health services are the major sources of information about HIV/AIDS, PLWHA and stigma reduction initiatives. Proximity and contact with health services will determine the degree of access to HIV transmission knowledge and familiarity with HIV treatments and care, each of which is associated with the extent to which participants endorse stigma.
Female heads of household who score high **on empowerment are less likely to endorse stigma**	Empowerment is operationalized as going against the social norm of male dominance in household level decision making. Based on the nature of power and social norms theory, we presume that once an individual rejects a powerful negative social norm s/he is less likely to endorse social norms that are harmful to others, such as the stigma of HIV/AIDS.

* The hypothesized relationships were expected to hold in unadjusted and adjusted analyses.

## Materials and Methods

Data from a general household survey conducted in Zambézia Province in 2010 under the *Ogumaniha*-SCIP Project provide a snapshot of HIV stigma within an established and generalized epidemic where a substantial response has been implemented. *Ogumaniha* means “united/integrated for a common purpose” in the local Echuabo language. SCIP stands for Strengthening Communities through Integrated Programming, a project implemented in Mozambique by a consortium of partners led by World Vision, Inc.

### Survey Background and Design

The *Ogumaniha*-SCIP Project commenced in Zambézia Province in late 2009. The project's baseline survey was conducted in late 2010 and recruited 3749 female heads of households in 259 randomly selected enumeration areas across 14 districts in Zambézia Province. Fourteen teams of 5 individuals, a team leader and four interviewers, collected data on a Zambézia-wide sample to provide province-wide estimates, as well as data in three focal districts to provide finer estimates from which to estimate changes over time [53]. The survey questionnaire included a module on HIV knowledge and attitudes towards PLWHA, including factors that might be associated with (and account for) HIV stigma. Interviews were conducted either in Portuguese or in one of 5 native languages of the province, and data were collected using mobile/cell phones. Details of the sampling procedures, identification of randomly selected enumeration areas and households, and the data collection and management process are published elsewhere [53]. Approximately 99.1% of all households approached agreed to participate in the survey. The study protocol was reviewed and approved by the National Committee of Bioethics for Health in Mozambique and the Institutional Review Board of Vanderbilt University [53]. Written informed consent was obtained for all study participants. A research team at Vanderbilt University was responsible for the collection of these survey data and is responsible also for maintaining the database. Information from the survey is de-identified, and there is no mechanism to re-link data back to individual participants.

This report is based on an analysis of these de-identified data. Ethical approval for this secondary data analysis study was provided by the Vanderbilt University Institutional Review Board (IRB#121003) who deemed that the study did not meet criteria for human subjects research. The analysis did not involve intervention or interaction with a “human subject” or access to identifiable private information.

### Stigma Measurement

Stigma items were adapted from a questionnaire used in a study conducted by Pulerwitz et al [11], [54]. The questionnaire lists 15 items reflecting attitudes, beliefs and behaviors that a respondent endorses at varying levels of intensity (on a 4 point Likert scale from ‘strongly disagree’ to ‘strongly agree’). The statements reflect labels and stereotypes that devalue and reduce a person with HIV to a tainted and socially undesirable status [14]–[16], [55] as well as specific discriminatory actions against PLWHA. Item exemplars include, “A person who has AIDS should not be allowed to work with other people to protect the people who don't have AIDS”, “AIDS is a punishment for bad behavior”. Factor analysis techniques were used to derive two dimensions of stigma (negative labeling & devaluation stigma and social exclusion stigma) and related scales (see statistical methods below). The modifications were mainly to make the items less about truck drivers but more about being a head of household. The Cronbach's alphas for the modified measure (i.e., α>0.70 for both stigma scales) were comparable to those reported by Pulerwitz et al. (e.g., α = 0.76 for the combined 15-item scale) [11], [54], indicating the measure was of acceptable reliability in this context as well.

### Main Correlates

*HIV transmission knowledge* was measured by the number of correct HIV transmission routes and ways to prevent them that a respondent was able to provide. These are domains of knowledge that are typically covered in public health education campaigns as well as in targeted health education programs conducted in both health centers and community settings. The focus was on adult-to-adult transmission and mother-to-child transmission routes and potential transmission via casual contact, making a total of 5 domains of knowledge. For example, “In what ways can one adult man or woman transmit HIV to another man or woman? How can HIV transmission from mother to child be prevented?” Interviewers were instructed to only record the number of correct responses for each potential HIV transmission event. Participants were not provided with a menu of potential responses to choose from and so had to provide the responses that they knew independent of the survey. A correct response for adult-to-adult transmission, for example would be unprotected vaginal, anal and oral sex, through needle sharing, blood transfusion, accidents in health settings. The interviewer selected one of 3 response options: 0 = None, 1 = One correct response and 2 = Two and more correct responses. A summative score (range: 0–10 points) was generated such that higher scores indicated higher (and better) knowledge.

*Legal rights certitude* was assessed via four questions about the extent to which the household has adequate and reliable access the traditional and modern legal/justice systems and have certitude of fair treatment should they need to resort to these systems. An exemplar item is “Does your household have access to the modern (state) legal system (court or tribunal) if you should need it?” Also, “Would you expect to be treated fairly by the modern (state) legal system?” A summary score from the Yes/No responses was generated and normalized to range from 0 to100.

*Familiarity with HIV infection* was assessed through self-reported awareness of people who are HIV+ and those in receipt of treatment for HIV/AIDS (i.e., relatives and friends) and own experience of HIV infection. Direct experience of HIV infection has been shown to moderate negative attitudes towards PLWHA [13] and this is consistent with findings about other socially stigmatized conditions like mental illness [2], [20].

*Belief about the efficacy of ART* was assessed through 2 questions about belief in the efficacy of ART, e.g., “Do you think antiretroviral treatment helps people with HIV to be healthier? Do you think alternative treatments available in the community or from traditional healers can help people with HIV?” Each item was treated as a distinct binary variable in the analyses since these are not mutually exclusive beliefs.

### Control Variables

We controlled for standard demographic variables of age, education, marital status, religion, contact with healthcare services, distance of place of residence from the clinic/health facility, and district of residence. Several districts in Zambézia are isolated (i.e., their main government health facilities are in remote rural locations). For the purposes of this study, the 14 districts were classified into two groups according to isolation of main government health facilities from the provincial capital of Quelimane. Isolation generally impacts the degree of coverage of public services, including healthcare and modern legal services. Other potential confounders considered were perceived risk of HIV infection, healthcare access/contact, social integration and empowerment as they reflect perceptions, behaviors and community level functioning that are likely to shape attitudes and behaviors towards PLWHA.

*Perceived risk of HIV infection* was assessed by asking: “What are the chances you might become infected with HIV?” Responses options were coded as follows: 1 = No chance, 2 = Small chance, 3 = Good chance and 4 = Already infected. Non-response or responses of “Don't know” were also recorded. Perceived risk was treated as a categorical variable with non-responses and “Don't know” responses collapsed into a single category.

*Healthcare services access/contact* was assessed by asking about three different healthcare systems that participants could utilize: government health centers or hospitals, private pharmacies and traditional healers. Each item was treated as a distinct binary variable in the analyses. The frequency of visits to each system was not estimated due to data reliability issues.

*Voluntary counseling and testing (VCT)* contact was assessed among participants who, in a separate question, reported that they were aware of VCT as follows: “Have you received voluntary counseling and testing (VCT) in the past 6 months? Have you ever received voluntary counseling and testing (VCT) at any time during your life prior to the last 6 months?” This was dichotomized into “ever used VCT” vs. “never used VCT.” At the time of the study VCT services were primarily offered in health facilities. However, the health facility contact variable captures purposes other than just (and different from) VCT. Secondly, undergoing VCT in itself (be it volitional action or otherwise, plus the experience at the VCT unit of the health facility) might be uniquely related to how VCT-using study participants answer stigma questions.

*Empowerment* was assessed through 10 questions about men vs. women's decision making roles in 10 domains of household level decision-making. Examples of domains of decision-making assessed are appropriate age to marry, family planning, administration of finances, seeking healthcare for pregnancy and farm/land chores. Response options were: (1) the men, (2) the women, or (3) both (men and women). Since these are primarily female heads of households in a context where decision power is generally skewed in favor of men, the scoring was re-coded to ensure that female dominance in households with adult males represented the greatest level of empowerment, while gender balance represented median empowerment. Such patterns of empowerment likely reflect unique household level norms about ‘power distance’ [56] in interpersonal relationships that might influence a head of household's willingness to stigmatize PLWHA. Some aspects of ART scale-up such as the prevention of mother-to-child transmission (PMTCT) services increase opportunities for women to be tested for HIV infection thus inadvertently making women vulnerable to community stigma, particularly in settings where gender inequality is rife. Hence, the association between community stigma endorsement and legal certitude might differ by degree of gender equality.

*Social integration* was assessed via 6 questions about the extent to which members of the household attend community development events, such as meetings about water and sanitation, the community health council, orphans and vulnerable children, agricultural and general community development. Attendance ranged from weekly to yearly or never. Public health education and general community strengthening occurs at such events in Zambézia Province as well as in other developing country settings. Attendance at such events is likely to shape household level attitudes and behavior towards PLWHA as well as level of HIV knowledge.

### Statistical Methods

Factor analysis using the principal component analysis (PCA) approach, with orthogonal varimax rotation, revealed two dimensions of stigma: negative labeling and devaluation (NLD) and social exclusion (SoE) (Table 2). The Cronbach's alphas for NLD and SoE were 0.74 and 0.73 respectively, explaining 94.7% of the variance. Cronbach's alphas were estimated to evaluate the internal reliability of the stigma constructs. Scales for each dimension were calculated by taking the mean value of non-missing items and then normalized to a 0–100 range. Univariate analyses (i.e., ANOVA) included survey-weighted proportion, median, and interquartile range by survey-weighted tertile of each stigma scale (Table 2). Tests of association with stigma scale (continuous) include Spearman's rank correlation (continuous) and rank sum test (categorical). Multivariable methods included linear models with robust covariance matrix estimates to correct for correlated responses from enumeration areas. Missing values of covariates were multiply imputed to prevent casewise deletion [57]. To account for possible non-linear associations, continuous variables were included in the models using restricted cubic splines [58]. Interaction effects were included to investigate modifying effects of ART efficacy and distance to clinic on stigma level. R-software 2.13.1 (www.r-project.org) was used for statistical analyses. Data are not publicly available, but analysis scripts are available at http://biostat.mc.vanderbilt.edu/wiki/Main/ArchivedAnalyses.

**Table 2:** Characteristics of the female heads of households by tertiles of NLD and SoE stigma scores^a^.

		NLD (n = 3219)				SoE (n = 3271)			
	Total	1^st^ Tertile	2^nd^ Tertile	3^rd^ Tertile	*P*-value	1^st^ Tertile	2^nd^ Tertile	3^rd^ Tertile	*P*-value
Sample N (%)	3323	(37.7)	(27.2)	(34.1)		(20.8)	(37.9)	(41.3)	
Age (years), median (IQR)	28 (23–36)	28	29	28	0.057	26	29	29	0.162
Education (years), median (IQR)	2 (0–4)	2	2	2	0.211	2	2	2	0.017
Distance from health facility (km), median (IQR)	6.2 (3.2–10.3)	6.6	6.2	6.2	0.012	5.8	6.2	7.6	<0.001
Geographically isolated district, % (95% CI)	56.4 (43.1, 69.7)	58.6	50.1	58.1	<0.001	69.0	45.8	55.5	0.704
Respondent understands Portuguese, % (95% CI)	42.0 (35.3, 48.8)	39.8	43.5	43.9	0.324	39.0	44.8	40.6)	0.011
Marital status, % (95% CI)					0.587				0.275
Married/Common Law	74.5 (70.9, 78.0)	73.5	74.1	76.0)		78.6	73.5	68.9	
Divorced/Separated	3.7 (1.6, 5.9)	2.6	7.5	2.4		5.6	3.2	2.7	
Single	17.0 (13.6, 20.5)	18.7	15.3	16.2		10.7	18.1	25.0	
Widowed	4.8 (2.6, 7.0)	5.2	3.1	5.4		5.0	5.2	3.4	
Religion, % (95% CI)^b^					<0.001				0.012
Catholic	47.7 (41.3, 54.0)	43.4	51.1	50.8		41.3	53.7	49.6	
Protestant	12.7 (9.4, 16.1)	18.2	8.7	8.5		14.8	13.4	12.1	
Evangelical and Pentecostal	16.6 (11.7, 21.6)	15.4	17.2	17.9		18.6	10.9	17.2	
Other Christian	4.4 (1.4, 7.4)	3.8	4.5	5.0		6.6	3.1	4.0	
Muslim	9.0 (5.4, 12.5)	10.5	7.6	7.8		10.5	9.3	6.8	
Non-Christian Eastern	2.1 (1.1, 3.1)	1.3	3.8	2.0		1.9	3.3	1.1	
Other	7.5 (5.0, 10.0)	7.4	7.0	8.0		6.2	6.2	9.1	
HIV knowledge (score) (n = 3219)	3 (0–4)	3	3	2	<0.001	3 (1–5)	3 (1–4)	2 (0–4)	<0.001
HIV infection of self, relative, and/or friend, % (95% CI)	12.5 (7.8, 17.3)	13.0	12.2	12.2	0.108	16.5	11.5	8.8	<0.001
Ever used VCT, % (95% CI)	20.3 (15.1, 25.6)	23.2	19.6	17.3	0.375	29.6	15.8	16.6	<0.001
Accessed health facility (%, n = 3219)	76.9 (72.6, 81.2)	77.7	81.1	72.8	0.939	80.8	75.0	72.6	<0.001
Accessed pharmacy, % (95% CI)	22.5 (15.6, 29.5)	21.9	21.3	24.2	0.212	23.9	24.3	19.9	0.051
Accessed traditional healer, % (95% CI)	45.9 (41.3, 50.4)	44.1	48.2	46.5	<0.001	50.0	43.1	44.3	0.008
Number of health services accessed, % (95% CI)	1 (1–2)	1 (1–2)	1 (1–2)	1 (1–2)	0.855	1 (1–2)	1 (1–2)	1 (1–2)	<0.001
Believes ART helps people with HIV to be healthier, % (95% CI)	31.5 (23.5, 39.4)	29.5	31.2	34.1	0.003	43.5	32.4	17.6	<0.001
Believes in alternative treatment for HIV, % (95% CI)	9.5 (7.3, 11.7)	7.9	10.5	10.9	0.002	9.0	11.1	6.8	<0.001
Perceived chance of becoming infected with HIV, % (95% CI)					0.073				<0.001
Don't know	47.8 (42.2, 53.3)	47.7	41.1	52.6		49.1	39.7	56.1	
No chance	24.5 (20.7, 28.4)	25.2	29.2	20.4		18.4	28.9	25.7	
Small chance	19.8 (16.2, 23.3)	18.3	24.1	18.6)		20.5	24.8	12.9	
Good chance	5.7 (4.0, 7.4)	6.6	4.0	5.7		8.2	5.6	3.9	
Already infected	2.2 (1.1, 3.3)	2.2	1.6	2.8		3.8	1.0	1.4	
Social integration (score), median (IQR)	89.3 (75–96.4)	89.3	87.5	89.3	<0.001	89.3	88	89.3	0.128
Empowerment (score), median (IQR)	50 (33.3–58.3)	50	50	50	<0.001	50	50	50	0.476
Legal rights (score), median (IQR)	100 (72.2–100)	100	88.9	83.3	<0.001	100	88.9	88.9	0.005
Income, median (IQR)	300 (0–700)	300	150	300	0.011	300	286	150	<0.001

[a] Continuous variables are reported as weighted estimates of median (interquartile range), with each observation being weighted by the inverse of the household sampling probability. Categorical variables are reported as weighted percentages, with each observation being weighted by the inverse of the household sampling probability. The 95% confidence intervals include precision estimates that incorporate the effects of stratification and clustering. Tests of association with stigma scale (continuous) include Spearman's rank correlation (continuous) and rank sum test (categorical).

[b] ‘Other Christian’ includes LDS Mormon and Jehovah's Witness. ‘Other’ includes Spiritual, Traditional Religions, and Agnostic or Atheist.

## Results

Characteristics of the study population are shown in Table 2, column 1. Of the 3749 female heads of household interviewed, 3323 (88.6%) had data on stigma. The mean NLD stigma score was 39 stigma units (SD = 17.6) and mean SoE score was 47 units (SD = 25.7), suggesting moderate to low intensity of community stigma. The median age of participants was 29 years old (interquartile range (IQR): 23–36 years) and did not differ by level of stigma. 50% of the sample had at least 2 years of education and fewer than 25% have more than 4 years of education, 78% reside in rural areas and 57% reside in isolated districts. Approximately three quarters said they were married (or in common law relationships) and 17% said they were single. Religious affiliations showed significant diversity, with 47% Catholics, 34% Non-Catholic Christians, 9% Muslims and about 10% other religions. Fewer than 50% of participants were fluent in Portuguese (official language). The average distance from the center of enumeration areas to the nearest public health facility was 6.2 km (IQR: 3.2–10.3 km). About 48% of the participants reported that they were not aware of their HIV infection risk, 25% were confident that they were not at any risk of HIV infection, while 25% thought they were at risk of being infected with HIV; 2% disclosed that they were already HIV positive. Disclosure of HIV status was higher (12%) in the subset that reported recent use of PMTCT services (results not shown).

About 12% were familiar with HIV infection through reported awareness of own or friend/close relative's HIV serostatus. Self-reported healthcare contact varied by type of healthcare: 76% reported lifetime use of public health facilities, 22% private pharmacy and 46% traditional healers. About one third believed that ART helps people with HIV to be healthier and about 9% think there is alternative treatment for HIV in the community or from traditional healers. Mean score for legal rights certitude was 100 points (IQR: 72.2–100). Participants who were excluded from the analyses because of missing stigma data (n = 426) did not differ by age, income, distance from clinic, HIV knowledge and other important variables of interest. However, they were less likely to be Catholic and to understand Portuguese.

In univariate analysis (Table 2, columns 2–8), NLD and SoE stigma share a number of correlates in these data, but they seem to be influenced by many other different factors and in a highly complex manner. In multivariable analyses the following relationships were observed:

### Endorsement of Negative Labeling and Devaluation (NLD)

Table 3 shows results of regressing NLD with survey responses. NLD was significantly (p<0.01) and inversely related to knowledge of HIV transmission routes, legal rights certitude and VCT access as hypothesized. For example, an HIV transmission knowledge score of 4 versus 2 was associated with a −1.47 unit change in NLD stigma score (95% CI: −3.37, 0.13), and a score of 6 versus 2 was associated with a −4.06 unit change in NLD scores (95% CI −6.20, −1.91), suggesting a negative trend. A legal rights certitude score of 100 versus 75 was associated with a −4.62 unit change in NLD stigma score (95% CI: −6.40, −2.85) but no significant difference in NLD scores was observed for legal rights certitude scores 50 versus 75.

**Table 3:** Multivariable linear (OLS) regressions of NLD and SoE stigma scores^a^.

	NLD		SoE	
	ß (95% CI)	*P*-value	ß (95% CI)	*P*-value
Age (per 5 years)	0.02 (−0.28, 0.32)	NS	0.08 (−0.37, 0.52)	NS
Education (per 5 years)	−0.29 (−1.80, 1.21)	NS	0.78 (−1.21, 2.77)	NS
Distance to Health Facility (per 1 km)	−0.13 (−0.31, 0.04)	NS	0.13 (−0.04, 0.30)	NS
Isolated district	−1.20 (−3.32, 0.93)	NS	−2.88 (−5.29, −0.46)	.019
Understands Portuguese	0.46 (−0.98, 1.90)	NS	0.18 (−1.67, 2.03)	NS
Marital Status		NS		NS
Married/Common Law (ref)	0		0	
Divorced or Separated	−1.50 (−4.74, 1.75)		1.04 (−3.40, 5.48)	
Single	−1.41 (−3.29, 0.46)		1.04 (−1.10, 3.18)	
Widowed	0.64 (−1.90, 3.19)		−2.73 (−5.64, 0.17)	
Religion		.002		.034
Catholic (ref)	0		0	
Protestant	−3.26 (−5.74, −0.78)		0.79 (−1.97, 3.55)	
Evangelical and Pentecostal	−0.31 (−2.32, 1.70)		−0.50 (−3.13, 2.13)	
Other Christian	−3.60 (−6.68, −0.52)		1.81 (−1.91, 5.54)	
Muslim	1.68 (−0.52, 3.88)		−4.17 (−6.80, −1.54)	
Non-Christian Eastern	−0.13 (−4.25, 3.97)		1.99 (−3.48, 7.46)	
Other	0.96 (−2.04, 3.97)		−1.50 (−4.56, 1.55)	
HIV knowledge score		<.001		<.001
0	−0.65 (−2.52, 1.23)		3.80 (1.70, 5.89)	
2 (ref)	0		0	
4	−1.47 (−3.07, 0.13)		−1.00 (−2.80, 0.81)	
6	−4.06 (−6.20, −1.91)		−3.52 (−5.89, −1.15)	
HIV infection of self, relative, and/or friend	2.04 (−0.42, 4.50)	NS	−3.17 (−5.78, −0.56)	.017
Ever used VCT	−2.45 (−4.22, −0.68)	.007	−0.39 (−2.58, 1.80)	NS
Accessed health facility	0.31 (−1.35, 1.97)	NS	−3.64 (−5.42, −1.86)	<.001
Accessed pharmacy	0.68 (−1.05, 2.41)	NS	1.18 (−0.71, 3.06)	NS
Accessed traditional healer	1.58 (0.30, 2.85)	.015	−1.06 (−2.63, 0.50)	NS
Believes ART helps people with HIV to be healthier	2.90 (1.14, 4.65)	.001	−6.94 (−9.12, −4.75)	<.001
Believes in alternative treatment for HIV	2.39 (−0.261, 5.04)	.077	0.07 (−2.64, 2.79)	NS
Perceived chance of becoming infected with HIV		.085		<.001
Don't know (ref)	0		0	
No chance	−0.98 (−2.79, 0.84)		4.54 (2.54, 6.54)	
Small chance	1.73 (−0.16, 3.62)		0.02 (−2.47, 2.52)	
Good chance	−0.69 (−3.72, 2.34)		0.68 (−2.67, 4.03)	
Already infected	−2.63 (−7.99, 2.74)		0.90 (−6.24, 8.03)	
Social integration (per 25 pts)	−0.45 (−1.45, 0.54)	NS	0.27 (−0.85, 1.39)	NS
Empowerment		<.001		<.001
25	3.94 (2.19, 5.70)		−4.44 (−6.38, −2.50)	
50 (ref)	0		0	
75	1.87 (0.25, 3.49)		−3.09 (−5.05, −1.14)	
Legal rights		<.001		NS
50	0.38 (−0.95, 1.71)		0.54 (−1.01, 2.08)	
75 (ref)	0		0	
100	−4.62 (−6.40, −2.85)		−0.05 (−2.03, 1.93)	
Income (per 500 MT)*	0.15 (−0.09, 0.39)	NS	0.18 (−0.50, 0.14)	NS

[a] NS = not significant (p>0.1). Because there was evidence (p<0.10) that the relationships with NLD and SoE were non-linear, HIV knowledge, legal rights and empowerment are fit using restricted cubic splines. About 7% of the variation in NLD scores can be predicted using the model (R^2^ = 0.066). The model also predicts about 9% of variance in SoE scores (R^2^ = 0.087).

* MT = Meticais (the currency of Mozambique).

**Figure 1:**
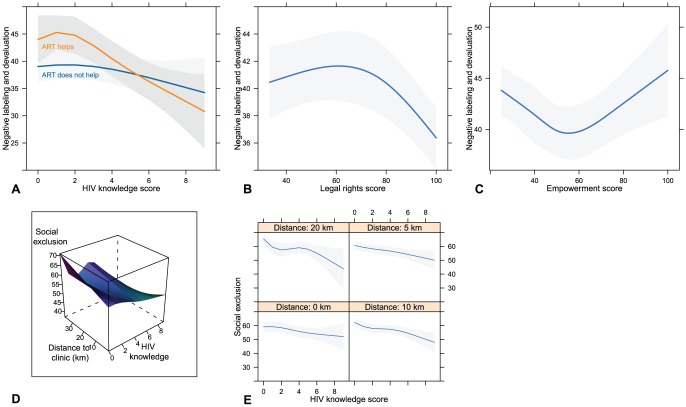
Stigma by HIV transmission knowledge, legal rights certitude, empowerment and distance to clinic.

Contrary to our hypothesis (Table 1), participants who believe that ART is helpful had on average of 2.90 greater NLD scores than those who do not consider ART helpful (β = 2.90, p = 0.001). The average difference in NLD scores between participants who contact traditional healers versus those who say they do not contact traditional healers was 1.58 stigma units (95% CI = 0.30, 2.85). The positive relationship with belief in alternative treatment for HIV (β = 2.39) was of marginal statistical significance (p = 0.077). Religion was associated with NLD scores: the significant difference in NLD scores was for Protestant vs. Catholics (β = −3.26; 95% CI = −5.74, −0.78) and Other-Christians vs. Catholics (β = −3.60; 95% CI = −6.68, −0.52). That is, Catholics are the reference category and had on average 3 times higher stigma scores than Protestants or Other- Christians. NLD had non-linear relationships with legal rights certitude, HIV knowledge, and empowerment (see Figure 1, Panels A, B and C). The marginal plot of NLD and HIV knowledge (differentiated by belief in ART efficacy) is shown in Figure 1, Panel A. The convex shape of the curves in panel A (with an inflexion at about point 4 of the knowledge scale) suggests that a significant drop in NLD endorsement only occurs among those who are highly knowledgeable about HIV transmission routes. Otherwise the stigmatizing attitudes of participants with low knowledge of HIV transmission routes are as negative as (if not worse than) attitudes of participants who were not able to provide a single accurate description of HIV transmission routes. This relationship is much clearer in the subgroup that believes that ART makes people with HIV healthier. The interaction between HIV knowledge and ART efficacy belief was also statistically significant (p<0.01), a partial support for the hypothesized interaction (Table 1). The moderating effect of ART efficacy belief was significant at HIV knowledge scores ≤4 units. The hypothesized interaction between HIV knowledge and distance to clinic on NLD stigma was not supported.

### Endorsement of Social Exclusion (SoE)

Table 3 also shows results of regressing SoE with individual level variables. SoE was significantly (p<0.01) and inversely related to knowledge of HIV transmission routes, HIV infection of self/relative/friend, ART efficacy belief, and living in an isolated district as hypothesized (Table 1). For example, independent of HIV transmission route knowledge, those who believe in ART's efficacy had on average a 7 unit drop in SoE stigma level than those who think ART does not make people with HIV become healthier (ß = −6.94; 95% CI = −9.12, −4.75; p<0.001). Compared with participants who said they did not know their risk of HIV infection, those who were definite that they were not at risk of infection scored 4.54 points more on the SoE scale (95% CI = 2.54, 6.54). We did not detect differences in the endorsement patterns of all participants who were in other self assessed infection risk categories versus participants who were risk unaware. HIV infection of self/relative/friend was inversely associated with SoE stigma (ß = −6.94; 95% CI = −9.12, −4.75; p<0.001). However, legal rights certitude was not related to SoE as hypothesized. The hypothesized interaction between HIV knowledge and distance to clinic was moderately supported (p = 0.209). Figure 1, Panels D and E indicate that the inverse association between SoE stigma and knowledge of HIV transmission was more pronounced among participants who live 10 km and 20 km away from the clinic than among those who live 0 km and 5 km away from the clinic. Religion was associated with SoE, but only among Muslims vs. Catholics—Muslims had on average 4 times lower SoE stigma scores than Catholics (ß = −4.17; 95% CI = −6.80, −1.54).

## Discussion

Factor analysis indicates the co-existence of the orthogonally distinct dimensions of negative labeling and devaluation (NLD) and social exclusion (SoE) stigma towards PLWHA. The NLD dimension of stigma is consistent with damaging labels and stereotypes of people living with HIV/AIDS that are detailed in the literature [6], [55]. One account noted as many as 290 descriptions of HIV/AIDS and PLWHA among communities in five Sub-Saharan African countries, most of which were negative or communicated harm caused by HIV [6]. Based on the mean score of each dimension of stigma, our sample of female heads of household expressed moderate-to-low levels of stigma. This endorsement of moderate-to-low intensity of stigma is consistent with findings from recent studies done elsewhere in the Sub-Saharan region [59]. Of the 11 statistically significant correlates, only one variable (HIV transmission route knowledge) has identical effects on both dimensions of stigma. Thus social exclusion and negative labeling may well operate as different mechanisms and so have orthogonality to them in observed conditions, i.e., an individual might despise but not necessarily discriminate against PLWHA.

However, higher knowledge about the transmission of HIV was related to lower stigma or reduced tendency to endorse negative attitudes and behavior towards PLWHA, regardless of the domain of stigma considered. This is consistent with our hypothesis (Table 1) and with literature from both resource-limited and higher income settings [60]–[63]. In other studies the inverse correlation with knowledge about HIV has been used as criterion for the construct validity of stigma scales [31]. Results from the baseline survey noted that the adult female participants in Zambézia Province have limited factual knowledge about HIV transmission and prevention [53]. As many as 20% of those surveyed in the province could not provide one correct mode of HIV transmission and about 50% stated they did not know how HIV is transmitted between adults or mother-to-child [53]. This might not be unique to Zambézia Province or Mozambique as similar observations have been made in some regions of neighboring South Africa [64]. These data also indicate that more knowledge is better at predicting low endorsement of community stigma, and that little knowledge might not be better than zero knowledge. Therefore, increased public education to improve knowledge of facts about HIV transmission might still be the way to reduce HIV stigma.

The interaction effect of distance from clinic and level of HIV knowledge on community stigma endorsement (see Figure 1, Panels D and E) further strengthens the role of outreach in HIV-related public health education in this setting. However, in separate analyses where models with and without the legal rights certitude variable were compared, the effects of distance from clinic were weakened by legal rights certitude (Results not shown). Furthermore, both the independent and interaction effects of ART efficacy beliefs (Figure 1, Panel A) suggest that knowledge of HIV transmission routes without strong belief in the efficacy of HIV treatment/therapies might not be enough to reduce stigma. These observations about the independent and moderating effects of legal rights certitude and ART efficacy beliefs suggest the importance of a broader scope for public health education than a sole focus on correct knowledge of HIV/AIDS etiology.

Overall, these relationships seem complex and dependent on the domain of stigma considered. For example, ART efficacy belief was associated with greater tendency to endorse negative labeling and devaluation (NLD) stigma and lower likelihood of endorsing social exclusion (SoE). The relationship with NLD stigma seems counterintuitive. However, similarly conflictive responses (if not ambivalence) to ART have been reported in other HIV stigma studies conducted in Sub-Saharan Africa, and attributed to the absence of a cure for HIV/AIDS [10], [65]. Roura and colleagues [65] observed that improvements in the health and functioning of persons on long-term ART engenders positive attitudes towards HIV disease among the non-infected, but does not change dominant (lay) attributions about HIV transmission and the belief that the HIV infected are fundamentally flawed and incapable of changing their risky and socially deviant behaviors. Theoretically, ART efficacy likely makes HIV infection invisible (i.e., purges externalized bodily characteristics by which to tag the infected), making it challenging to apply lay criteria for screening the HIV infected and exerting social control over them. As noted by Roura and colleagues [65] ART efficacy might create new bases for social anxiety about HIV infection in some settings. Elsewhere it has also been noted that, while there is an increasing willingness among health workers to treat HIV infected patients, high knowledge about HIV transmission routes coupled with poor infection control practices might inadvertently increase the fear of contagion among these health workers [66]. Mental illness literature, which has a lot to say about stigma, has shown that treatment efficacy does not guarantee elimination of stigma [67]–[71]. For example, the stereotype of dangerousness, that is rife in views of mental illnesses, has been shown to persist in attitudes towards the treatment experienced or those considered to have responded well to treatment for mental illness [67], [71]. Our data suggests the possibility that negative stereotypes might persist in the public mind despite increased knowledge of HIV prevention and treatment efficacy.

The relationship between perceived risk of HIV infection and stigma is unclear and weak. However, believing that one is at no risk of HIV infection was associated with the greatest intention to socially exclude PLWHA independent of all other factors. Among those who considered themselves to be at some risk of HIV infection, social exclusion stigma did not vary by intensity of perceived risk. Thus public education to improve awareness of the risk of HIV infection is needed. Another observed anomaly is that increased familiarity with HIV infection was associated with greater endorsement of negative labeling and devaluation but less willingness to socially exclude PLWHA. Given the negative association observed between legal rights certitude and negative labeling and devaluation, the inconsistent effect of familiarity with HIV infection could be due to difference in social norms about behavior vs. attitudes, i.e., overtly discriminatory behavior might not be socially accepted in this context. We cannot ascertain if such self-censorship is present outside of our research context, i.e., whether or not the difference observed in these data is an artifact of the survey interview itself. Since these are cross-sectional data and no comparable study of these factors has been done among female heads of households in Zambézia Province, we cannot confirm these observations. These and related issues need to be further investigated.

Capabilities such as empowerment agency and knowledge of legal rights are increasingly seen as critical for enhancing the ability of individuals and communities to move out of poverty and socio-political oppression [72]. Indeed some of the anti-stigma initiatives in Mozambique and elsewhere have prioritized clear definition and guarantees for the legal rights of PLWHA [32], [39]. Data on the impact of promoting greater awareness and protection of the legal rights of PLWHA or of strengthening generic legal institutions on community stigma are scarce. The legal system can influence community stigma by dictating acceptable and unacceptable conduct and expressions, means of redress available to victims of stigma, and punishments for offenders. One might, therefore, expect a negative relationship between these capabilities and endorsement of stigma towards PLWHA (see list of hypotheses in Table 1). However, no clear relationships between capabilities (i.e., legal rights certitude and empowerment) and stigma were observed in our data. Nonetheless, there was some support for our hypothesis that confidence in the household's access to the modern legal system and the likelihood of due process (i.e., legal rights certitude) is associated with lower endorsement of HIV stigma. The observed relationship was with negative labeling and devaluation but not with social exclusion stigma. We do not know if knowledge of HIV-specific statutes would have a different effect since such data were not gathered in the *Ogumaniha*-SCIP survey. Our data suggest that there could be added value in raising community awareness of the legal rights of PLWHA (and ways to exercise them) as part of health education strategies for reducing community stigma. The potential effects of knowing HIV-specific legal rights and that of confidence in the workings of the legal system on community stigma need to be further investigated. One behavioral and emotional anti-stigma response that has been associated with legal rights knowledge is righteous anger among people who have (or identify with people with) a stigmatized condition. Watson and colleagues (2007) observed that people who considered negative stereotypes of mental illness to be illegitimate and had an intact sense of self-worth (i.e., had high self-esteem despite community stigma) tended to externalize their disdain for community stigma through expressions of righteous anger rather than accept community stigma and blame themselves for the stigmatizing condition. In our study NLD stigma endorsement is a close approximation to agreeing with negative stereotypes. Thus the relationship between legal rights certitude and NLD stigma is likely to be moderated by perceived legitimacy of community stigma. Such a moderation model needs to be examined in future studies so that the impact of human rights awareness on HIV stigma can be specified much more clearly than was done in the present study.

Demographic variables also showed important effects. For example, the influence of religious affiliation seems to depend on domain of stigma considered: the major difference seems to be between the self-reported attitudes and behaviors of Muslims and Non-Catholic Christians. Compared to Catholics, Muslims were significantly less likely to endorse the social exclusion of PLWHA, but more likely to endorse negative labeling and devaluation (Table 2). Compared to Catholics, Protestants and those in the Other Christian category (which includes Latter-Day Saints Mormon and Jehovah's Witness) had less tendency (i.e., lower mean score) to endorse the negative labeling and devaluation of PLWHA. Although those self-reporting affiliation to Muslim and other religions (which includes spiritual, traditional, agnostic and atheist religions) had higher NLD scores than Catholics, these differences were not statistically significant (Table 2). Religious social norms likely shape HIV stigma in this context. The difference made by type of religion could be seen as indicating the importance of tailoring anti-stigma interventions to the religious affiliations of target groups. There might be added value in exploring the potential for interfaith strategies for stimulating community-wide dialogue about HIV stigma and ways to address it over single-faith strategies.

In other studies, the interaction between stigma and perceived risk of HIV infection has been shown to significantly influence uptake and engagement in HIV services [50]. In this study, contact with voluntary counseling and testing facilities (VCT) was negatively related to NLD but unrelated to SoE, while contact with health facilities in general was negatively related to SoE but unrelated to NLD stigma. The impact of stigma on services uptake might be dependent on the domain of stigma being considered.

The model R^2^ statistics were ≤10%, indicating that a significant proportion of the variance in stigma observed in this sample is not explained by the ‘theory consistent’ predictors explored in this study. Alternatively, there was not enough variability in the levels of stigma assessed through this survey. The potential for measurement error needs to be further investigated. There is a possibility that a different stigma instrument, perhaps with more and different items, could have yielded other and more precise domains of stigma. The challenges of finding suitable and validated stigma scales are acknowledged in the literature and present general limitations to stigma research. The stigma module (embedded in the *Ogumaniha*-SCIP baseline survey) was intended to generate data to describe the nature of the cultural context within which people live with HIV/AIDS in rural Mozambique. HIV stigma reduction is not a core priority of the *Ogumaniha*-SCIP baseline survey and so would not accommodate lengthy stigma instruments and complex validation study designs. Nonetheless, these data could be the beginning of a process of tracking changes in attitudes over time (part of surveillance monitoring) similar to the General Social Survey in the USA [70] and other settings [73]. Our sample only included female heads of household. Therefore, our findings might not apply to male heads of household. Gender differences in stigma expression and expectancy and the likelihood of ambivalence to ART efficacy need to be investigated further. We could not distinguish the manifestation of NLD and SoE stigma among HIV positive vs. HIV negative individuals because we had no reliable data on the serostatus of study participants. However, the possibility of distinguishing anti-stigma interventions by serostatus needs to be investigated, particularly based on how NLD and SoE stigma are related differently to HIV familiarity and perceived risk of HIV infection variables as well as proxies of contact with the health system.

## Conclusions

Our analysis shows the complex relationships among increased HIV/AIDS knowledge, perceived ART efficacy, legal rights certitude and demographic characteristics, and stigma towards PLWHA in rural Mozambique. Part of the complexity stems from the potential duality of rejection and acceptance in people's attitudes and behavior, and the fact that the same knowledge, beliefs and environmental factors impact rejection (NLD stigma) and acceptance (SoE stigma) tendencies differently. Prejudice literature, as well as findings about mental illness stigma, suggests that stigma reduction is one of several potential outcomes of stigma reduction interventions, including a change from overt to increasingly subtle forms of stigma [17], [63]. Creative anti-stigma strategies are needed that take advantage of the observed disjuncture in the labeling process of stigma, i.e., the duality of negative labeling and devaluation, on the one hand, and social inclusion on the other. Effective stigma reduction strategies at the community level may have to be domain specific, with emphasis on the enduring effects of negative labeling and devaluation.
